# Spatial analyses of wildlife contact networks

**DOI:** 10.1098/rsif.2014.1004

**Published:** 2015-01-06

**Authors:** Stephen Davis, Babak Abbasi, Shrupa Shah, Sandra Telfer, Mike Begon

**Affiliations:** 1 School of Mathematical and Geospatial Sciences, RMIT University, Melbourne, Victoria 3001, Australia; 2 School of Biological Sciences, University of Aberdeen, Zoology Building, Tillydrone Avenue, Aberdeen AB24 2TZ, UK; 3 Department of Evolution, Ecology and Behaviour, University of Liverpool, Crown Street, Liverpool L69 7ZB, UK

**Keywords:** epidemiology, mathematical model, field vole, *Microtus agrestis*, graph, dissimilarity measure

## Abstract

Datasets from which wildlife contact networks of epidemiological importance can be inferred are becoming increasingly common. A largely unexplored facet of these data is finding evidence of spatial constraints on who has contact with whom, despite theoretical epidemiologists having long realized spatial constraints can play a critical role in infectious disease dynamics. A graph dissimilarity measure is proposed to quantify how close an observed contact network is to being purely spatial whereby its edges are completely determined by the spatial arrangement of its nodes. Statistical techniques are also used to fit a series of mechanistic models for contact rates between individuals to the binary edge data representing presence or absence of observed contact. These are the basis for a second measure that quantifies the extent to which contacts are being mediated by distance. We apply these methods to a set of 128 contact networks of field voles (*Microtus agrestis*) inferred from mark–recapture data collected over 7 years and from four sites. Large fluctuations in vole abundance allow us to demonstrate that the networks become increasingly similar to spatial proximity graphs as vole density increases. The average number of contacts, 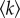, was (i) positively correlated with vole density across the range of observed densities and (ii) for two of the four sites a saturating function of density. The implications for pathogen persistence in wildlife may be that persistence is relatively unaffected by fluctuations in host density because at low density 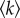 is low but hosts move more freely, and at high density 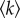 is high but transmission is hampered by local build-up of infected or recovered animals.

## 1. Introduction

There is growing interest among disease ecologists in elaborating contact networks in wildlife populations and the likely consequences for the spread of pathogens or parasites [1–8]. Theoretical studies have, in particular, shown that (i) pathogens tend to spread rapidly and easily on networks containing small numbers of highly connected individuals and (ii) if those highly connected individuals can be targeted for either vaccination or removal then it becomes easier to prevent an outbreak or mitigate its effects [9]. Hence, a focus of recent studies has often been the detection of high individual heterogeneity in numbers of contacts, and whether characteristics such as age, sex or size might be used to predict which individuals have the highest numbers of contacts. By contrast, analyses that quantify spatial constraints on who has contact with whom have largely been absent, even though spatial constraints are capable of critically affecting infectious disease dynamics [10,11]. Craft *et al*.'s [3] study of contacts between prides of Serengeti lions is an exception, but the approach is highly tailored to the unique datasets arising from the Serengeti Lion Project. Here we propose two approaches: (i) graph dissimilarity measures that quantify how close an observed network is to being a proximity graph (i.e. one in which the edges of a network are purely determined by the spatial arrangement of the nodes) and (ii) maximum-likelihood approaches to quantify spatial constraints and judge between competing network models. Finally, we fit a class of good-get-richer network models [12,13] that incorporate and quantify both spatial constraints and individual heterogeneity. We apply all of these approaches to a set of 128 contact networks constructed from a large mark–recapture dataset on field voles (*Microtus agrestis*) collected over a 7-year period.

The role of spatial constraints in determining the dynamics of infectious disease is particularly pertinent for territorial animal populations, where the question arises as to whether territoriality offers a level of protection from disease outbreaks. In territorial populations, two animals may normally have contact only if their home ranges overlap, with exceptions arising from rare long distance dispersal events or nomadic individuals. Infectious diseases of such populations must overcome what is effectively a spatial barrier if they are to spread and persist; transmission must occur frequently enough to escape local build-up of infected and recovered animals and avoid fade-out. Such spatial effects can be understood to slow an epidemic down in the same way as clustered contact patterns, or the presence of short loops in networks [14]. The effect is that infectious individuals are more likely to have neighbours that are in the recovered state, and also neighbours that are infected, which they may then ‘compete’ with for the few remaining susceptibles. In such circumstances, the use of epidemiological theory based on random mixing of hosts overestimates the ability of the pathogen to spread, undermining, for example, the use of the basic reproduction number, *R*_0_, to predict threshold conditions for outbreaks. This has been well illustrated for the occurrence of epizootics of sylvatic plague (*Yersinia pestis* infection) in populations of great gerbils (*Rhombomys opimus*) in Central Asia [15,16].

More generally, the concern of epidemiologists with contact networks can be interpreted as an acknowledgement that the transmission of infection occurs at the individual level but its epidemiological consequences are played out at the population level, and that it is important to understand how the two are related [17]. In particular, it would be valuable to understand whether certain contact structures translate into the canonical density- and frequency-dependent transmission functions or into variants of these and intermediates between them that have been proposed (e.g. [18,19]). Here, therefore, we also explore these connections, as data from the same field vole system have previously been analysed to identify and interpret the transmission function for cowpox virus transmission that best fits population-level data [18].

## 2. Material and methods

### 2.1. Trapping data and field sites

The study took place in Kielder Forest, a man-made spruce forest occupying 620 km^2^, situated on the English–Scottish border (55°13′ N, 2°33′ W). Field voles inhabit grassy clear-cuts that represent 16**–**17% of the total area, but are completely absent from forested areas that isolate the clear-cuts. Clear-cuts range in size from 5 to 100 ha. Field vole populations at Kielder fluctuate cyclically with a 3**–**4 year period [20]. Voles were trapped in four similar-sized clear-cuts, in two areas of the forest approximately 12 km apart, between May 2001 and March 2007. In the Kielder catchment, Kielder Central Site (KCS) and Plashett's Jetty (PLJ) are situated 4 km apart. In the Redesdale catchment, Black Blake Hope (BHP) and Rob's Wood (ROB) are 3.5 km apart. Thus, these four populations were far enough apart, with sufficient forest between them, to be considered as effectively independent replicates.

Populations were trapped in ‘primary’ sessions every 28 days from March to November, and every 56 days from November to March. Each site had a permanent 0.3 ha live-trapping grid consisting of 100 Ugglan Special Mousetraps (Grahnab, Marieholm, Sweden), in optimal habitat dominated by *Deschampsia cespitosa*, *Agrostis tenuis* and *Juncus effusus*. Traps were set at 5 m intervals and baited with wheat and carrots. Traps were pre-baited with a slice of carrot and a few grams of oats 3 days before each trapping session, set at approximately 18.00 on the first day and checked five times (referred to ‘secondary’ sessions; a ‘primary session’ thus refers to a cluster of five ‘secondary’ sessions) at roughly 12 h intervals starting and ending at dawn and dusk, respectively. Individual animals were identified using subcutaneous microchip transponders (AVID plc, East Sussex, UK) injected under the skin at the back of the neck. Mass, sex and reproductive status (assigned according to the external appearance of reproductive organs) were recorded at the time of first capture in each primary session. Estimates of total population size were derived in program MARK using Huggin's closed capture model within a robust design [21].

We formed networks from the mark–recapture data by supposing each vole trapped was a node of a spatial network. A spatial location for each node was determined as the average position of the traps it was caught in, with trap location weighted by the number of times the vole was caught in that trap. This follows the practice of other wildlife epidemiologists working with similar data [1–8]. An edge was inserted into the network whenever two voles were caught in *at least one* common trap over the primary trapping sessions being considered. Thus, multiple edges are avoided and the degree of a node (the number of edges connected to it) can be interpreted as the number of unique contacts a vole has over the period of observation. There is potentially an important difference between the rate of contact that includes repeated contacts between the same individuals and the rate at which new contacts are made, and we note that it is also possible to form networks that do include repeated contacts and hence multiple edges. We note that there are a number of other constructions possible from these data that would form slightly different sets of networks. One could be more ‘strict’ about what constitutes indirect contact by having weighted edges (number of traps in common) and then thresholding on the weights to produce simple networks. There are also many ways to define the spatial location of the node set, using either a subset of the trap locations or a different measure of central tendency.

Exploratory work indicated that the contact networks based on a single primary trapping session have no, or very few, edges when the vole densities are low. So that we could consider how the networks varied with population density we considered combining trapping sessions to form the networks. Voles are sometimes seen in only one trapping session and then never again, but for much of the time a vole is seen in two or more consecutive trapping sessions. Two trapping sessions hence provided a better basis to define a geographical location for each vole (more trap locations) and defined more edges so that even at the lowest vole densities the networks had a reasonable number of edges. When three (or more) trapping sessions are combined (see the electronic supplementary material for a comparison) an edge can represent anything from a vole visiting a trap two months after another did, or a vole visiting a trap the next night. We therefore chose to form networks from pairs of consecutive trapping sessions. At each site, there were 64 trapping sessions, and hence 32 networks were formed for each of the four sites, with each network derived from a consecutive pair of primary trapping sessions and each trapping session appearing in only one network. This set of 128 networks represents a range of contact patterns, occurring at different times of the year and at different vole densities.

### 2.2. Basic graph measures

For each of the 128 contact networks, we calculated the mean degree, 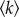 to estimate the average rate of contact, and the coefficient of variation (CV) of the degree distribution. We tested whether there was more support from the data for a linear, *a* + *bN*, versus a power relationship, *a* + *cN*^1/*α*^ (*α* > 1), between 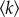 and vole density to see if there was evidence that contact rate was a saturating function of density. We used adjusted *R*^2^ to account for the difference in the number of parameters between the competing models.

### 2.3. Proximity graph dissimilarity measures

The networks inferred from the mark–recapture data consist of a point pattern of nodes, and a set of undirected edges. As a measure of how spatially constrained a contact network is, we propose below two normalized measures of dissimilarity. They are both counts of the edge differences between the observed contact network and a proximity graph constructed from the point pattern of nodes of the observed contact network [22]. A proximity graph has an edge between two nodes if particular geometric requirements are met, and hence are entirely induced by the underlying point pattern. Here we consider proximity graphs based on the geometric requirement that two nodes are within some set distance, *ɛ*. Varying this threshold distance produces a family of graphs, indexed by *ɛ*. Low dissimilarity values then indicate that the observed graph is close to what would be expected if contacts between individuals were made purely on the basis of proximity, as measured by Euclidean distance.

More formally, let *P_*ɛ*_* be the proximity graph *P_*ɛ*_* = {*V*, *L*, *f*} where *V* is the set of nodes, *L* the set of edges and a mapping *f* : *L* → *V* × *V*, where *f*: *v_i_* ∼ *v_j_* if *s_ij_* ≤ *ɛ*, and where *s_ij_* is the Euclidean distance between nodes *i* and *j* belonging to *V*. We next denote the adjacency matrix for *P_*ɛ*_* by A*, having elements 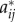 which take a value of 1 when an edge exists between node *i* and node *j* and 0 otherwise. Also, we denote the observed network of interest by *G*, having adjacency matrix **A** with elements *a_ij_* and distance matrix **S** (the matrix of distances, *s_ij_*). We can then define2.1
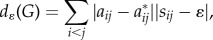
where |·| denotes absolute value. The formula counts differences between the adjacency matrix of the observed graph and the adjacency matrix of the proximity graph *P_*ɛ*_*. A difference indicates either that an edge in *G* is missing from *P_*ɛ*_* or an edge in *P_*ɛ*_* is missing from *G*. Edge differences are weighted by the linear factor |*s_ij_* − *ɛ*| so that an edge missing from between two nodes that are very close together contributes more to the dissimilarity measure than an edge missing from two nodes that are about *ɛ* distance away. Similarly, edges longer than *ɛ* that are in *G* but not in *P_*ɛ*_* contribute more the longer they are. That is, as long edges are (by definition) not a feature of this type of proximity graph, their presence in the observed graph represents a strong dissimilarity.

Next, we normalize the weighted sum of differences, because such a sum will be affected by the size of the network (the number of elements of the adjacency matrix increases as *n*^2^) and we would like to make comparisons between graphs of unequal size. We do so by dividing by the sum of weights, the |*s_ij_* − *ɛ*|, for all possible pairs of nodes. This is equivalent to counting up the weighted differences between the proximity graph and its complement (which has the same set of nodes as *G* with the same spatial arrangement and has an edge between two nodes if and only if the corresponding edge is missing in *G*). This gives2.2
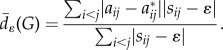


Let the value of *ɛ* that minimizes 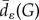 be ɛ*. This gives 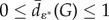 as a simple measure of dissimilarity between the observed spatial graph, *G*, and the family of proximity graphs induced by the observed point pattern of *G*. For simplicity of exposition, we refer to 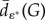 as *D*.

There are clearly other possible choices for the weighting used in equation (2.1), the determination of ɛ* and the normalization. For example, an unweighted version of *D*, which we will denote *D_u_*, is given by finding the value of *ɛ* that minimizes2.3
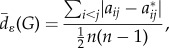
where the denominator in equation (2.2) becomes the number of *possible* edges in the graph. *D_u_* has the advantage of having a very simple interpretation. It is the fraction of entries in the adjacency matrix that are ‘wrong’ in the sense of being different to the corresponding entry for the closest proximity graph (closest being defined by the value of *ɛ* that minimizes equation (2.3)). In either case, weighted or unweighted, a value of 0 indicates that *G* is in fact a proximity graph where the topology is entirely determined by the spatial arrangement of the nodes.

As a non-network measure of how spatially restricted the voles were, we used trap locations to calculate a distance deviation for each node (vole) in each network (see the electronic supplementary material for details), representing the observed spatial variance of an individual vole over the two trapping sessions. We investigated how the distribution of distance deviation changes with population density, and how the average distance deviation for three categories of voles (large male, small male and female; see below) changes with population density.

### 2.4. Model fitting

The second approach we propose is to fit simple models for the rate of contact between two voles given the distance between them to the observed binary edge data (presence or absence of an edge). The simplest model (herein referred to as model 0) proposes that the rate of contact, *k_ij_*, between any two voles in the network (node labels *i* and *j*) is constant; this is equivalent to the random-mixing assumption where every vole is equally likely to make contact with every other vole:2.4
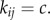
We set the time unit to be the time period over which the data used to construct the network was collected, so *c* is to be interpreted as the number of contacts per sampling period.

To quantify whether and to what degree spatial constraints play a role in determining the contact rates of voles, we also considered the model2.5
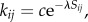
where *s_ij_* is the Euclidean distance between nodes *i* and *j*, and *c* and *λ* are constants. We will subsequently refer to the model represented by equation (2.5) as model 1. The magnitude of *λ* determines the scale over which the spatial constraints operate, such that for positive values the contact rate between two voles will decline in a negative exponential manner as the distance between them increases. A value of *λ* close to 0 indicates support for a random network. By contrast, high values of *λ* indicate that the probability of an edge (contact) declines sharply with the distance between nodes. For example, recalling that traps are spaced 5 m apart, a *λ* value of 2 indicates that each additional 5 m between the locations of two voles decreases the rate of contact by an order of magnitude (approx. exp(−2)).

Finally, to investigate whether there was support for individual heterogeneity in our data, we considered two additional models (referred to as model 2 and model 3, respectively) that belong to the class of good-get-richer network models proposed by Caldarelli *et al*. [12]. The first of these models incorporates both spatial constraints and individual heterogeneity in the rate of contact by allocating each individual a ‘fitness’ value (denoted *x_i_*). In this context, these values represent the tendency for an individual to apparently seek out or avoid contact. Rather than estimating individual ‘fitness’ values, we defined subgroups of animals based on size and sex (these were large males, small males and females) and allocated a value to each subgroup, respectively, denoted by subscripts M, m and F. We hence considered the model2.6
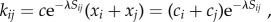
where 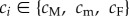. This model, referred to as model 2, allows the different groups to behave differently with respect to an overall propensity for contact, i.e. for a fixed distance *k_ij_* ∝ (*c*_*i*_ + *c_j_*). However, the inhibiting effect of distance on this propensity for contact is the same for all possible pairings {*i*, *j*} since *λ* is a constant. We hence also considered2.7
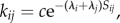
where 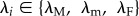, as model 3. This model allows the groups to vary in how inhibited contacts are by distance.

For all four models, the probability of observing an edge between hosts *i* and *j*, denoted by *p_ij_*, can be related to the rate of contact by assuming that the number of contacts between *i* and *j* over the period of observation has a Poisson distribution with intensity *k_ij_*. The probability of observing at least one contact is then 1 minus the zero term in the Poisson distribution, giving,2.8
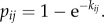
We fitted model 0 and model 1 in R [23] using a binary generalized linear model (GLM) with a ‘complementary log–log’ link (having functional form log(−log(1 − *p*))). In the case of models 2 and 3, these cannot be fitted using GLM although a roughly equivalent model is possible (see the electronic supplementary material).

To fit models 2 and 3, we further define *a_ij_* as an element of the adjacency matrix for the contact network of interest, *n* as the size of the network, *Ω* = {(*i*, *j*)|*a_ij_* = 1}, and *Ω*′ as the complement of *Ω*. Model parameters can then be estimated using maximum-likelihood where the likelihood is2.9
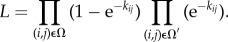


We used the simulated annealing algorithm that is included in the function *optim* in R [23] to maximize the log likelihood [24]. We note that when comparing model 1 with model 2, it can be seen that model 2 degenerates to model 1 when *c*_M_ = *c*_m_ = *c*_F_ = 1/2*c*, and similarly model 3 degenerates to model 1 when *λ*_M_ = *λ*_m_ = *λ*_F_ = 1/2*λ*. We took advantage of this by using the optimal parameter values from model 1 to set initial values for the simulated annealing algorithm (when fitting model 2 and model 3). For some networks with low numbers of individuals, the algorithm used by the function *optim* failed to converge to a lower likelihood or the model was a worse fit.

We fitted models 0–3 to each of the 128 networks separately. We also pooled the data and combined the 128 networks to test for effects of vole density and site on the slope and intercept of a binary GLM. In all cases and all statistical fitting methods, we used Akaike's Information Criterion corrected for small sample sizes (AICc) to judge the relative performance of the models. All analyses were conducted using the statistical software package R [23].

Finally, model 1 was fitted to equivalent random networks to better understand how estimates of *c* and *λ*, and especially *λ*, might be affected by the way the networks were constructed. This is a concern because the spatial location of each vertex and the edges drawn between the nodes are derived from the same data (the location is the average of the trap locations a vole was caught in; edges are inferred when two voles are caught in the same trap). This was done for only one of the observed networks, KCS, September–October 2003 (a medium-sized network having 73 nodes). To generate ‘equivalent’ random networks comparable to an observed network consisting of *n* nodes and *m* edges, we generated equivalent random trap data for *n* voles. The actual trap data take the form of an incidence matrix, with 100 columns corresponding to the 100 trap locations, and the number of rows equal to *n*. It was not unusual for individual voles to be found in the same trap more than once and so the entries of the incidence matrix are the number of times a particular vole was caught in a particular trap. In generating equivalent random trap data, the sum of each row (the number of voles) was conserved but the trap locations for the voles were randomized. The equivalent random networks were generated from the equivalent random trap data in the same way as the observed networks were generated from the actual trapping data.

## 3. Results

### 3.1. Vole contact networks

**Figure 1.**
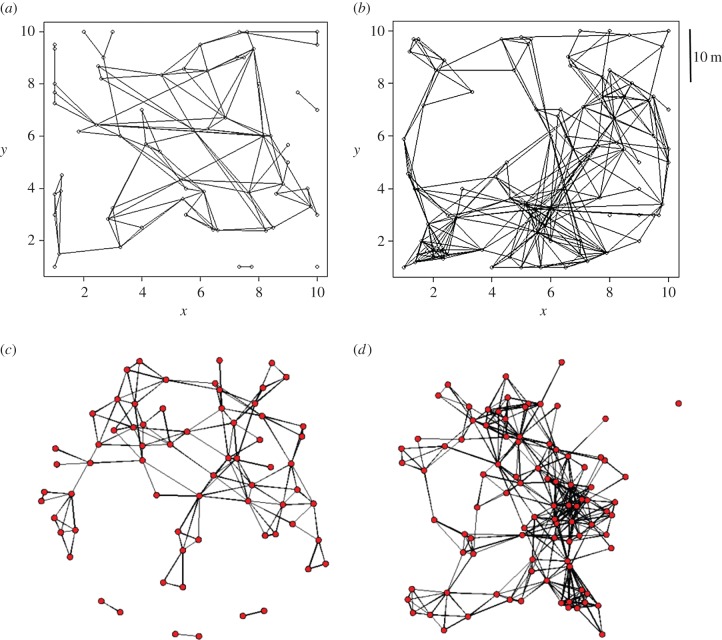
Spatial and non-spatial plots of two of the 32 contact networks inferred from trapping sessions conducted at the Kielder Site (KSC) during (*a*) winter (from 13 November 2001 to 20 January 2002) and (*b*) summer (from 28 June 2002 to 26 July 2002). The non-spatial versions of these networks, (*c*) and (*d*), respectively, are produced in the software package R where there is an attempt to more clearly display the structure of the networks by minimizing the number of edge-crossings. (Online version in colour.)

The 128 networks varied dramatically in size from 11 to 264 nodes. The networks were typically well connected in the sense that there was usually a single large component and a small number of isolated nodes or very small components (see electronic supplementary material, figure S1). Two examples, both from KCS, are shown in figure 1, one derived from trapping in winter from 13 November 2001 to 20 January 2002 and the other from trapping in summer from 28 June 2002 to 26 July 2002 when vole density was higher. The same data are displayed as spatial graphs (figure 1*a*,*b*, each node has coordinates) and non-spatial graphs (figure 1*c*,*d*, spatial locations are ignored).

### 3.2. Basic graph measures

**Figure 2.**
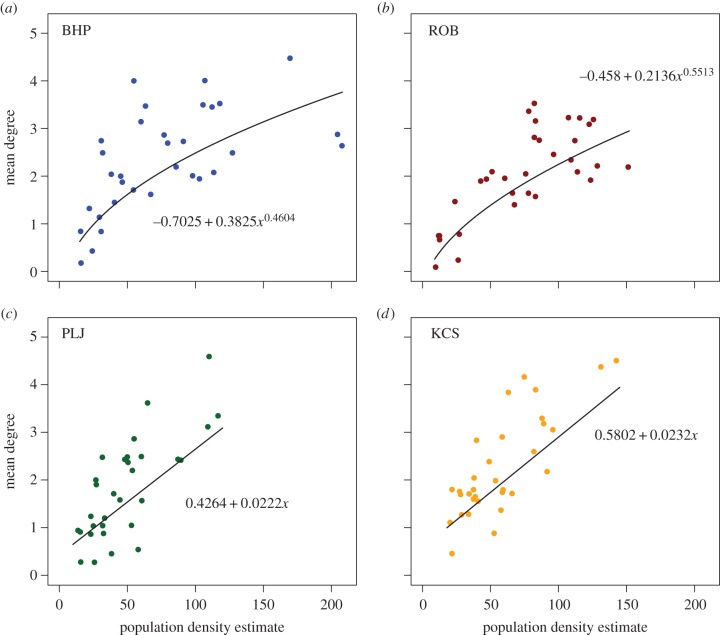
The mean degree of each of the 32 networks, from each of the four sites, plotted against estimated population density. A linear function and a power function were fitted to the data separately for each site. In (*a*,*b*), for BHP and ROB, a power function was a better fit (adjusted *R*^2^ values were, respectively, 0.669 versus 0.427 and 0.778 versus 0.642), while in (*c*,*d*), for PLJ and KCS, a linear function was the better fit (adjusted *R*^2^ values were, respectively, 0.783 versus 0.6689 and 0.7308 versus 0.6907). (Online version in colour.)

**Figure 3.**
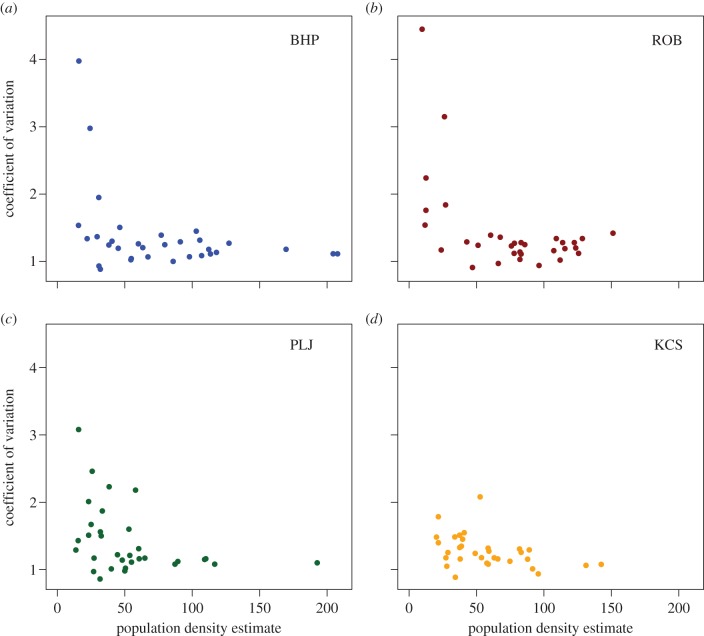
The CV (the standard deviation of the degree sequence scaled by the average degree) of each of the 32 networks, from each of the four sites, plotted against estimated population density. The CV tends to drop as population density increases. Consistent with the plots of mean degree shown in figure 2, the results for sites BHP and ROB appear to show a similar pattern to each other, as does the pairing PLJ and KCS. (Online version in colour.)

Scatter plots of the mean degree and the CV of the degree distribution, versus estimated vole density, are shown for all sites in figures 2 and 3. Mean degree (figure 2) was positively correlated with estimated population size. For two of the four sites, a power relationship (where 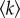 is a saturating function of density) performed better (as measured by adjusted *R*^2^) than a linear relationship. The scatterplots of the CV at sites BHP and ROB (figure 3*a,b*) show that for vole densities larger than approximately 50 voles per hectare, the CV drops to values not much larger than 1. For the other two sites, PLJ and KCS, the scatterplots show an absence of points in the upper right triangle of the axes, indicating high values of the CV are not observed at high densities. For all sites, the scatterplots indicate there is more heterogeneity in numbers of contacts at lower densities than at higher densities (figure 3).

### 3.3. Proximity graph dissimilarity measures

**Figure 4.**
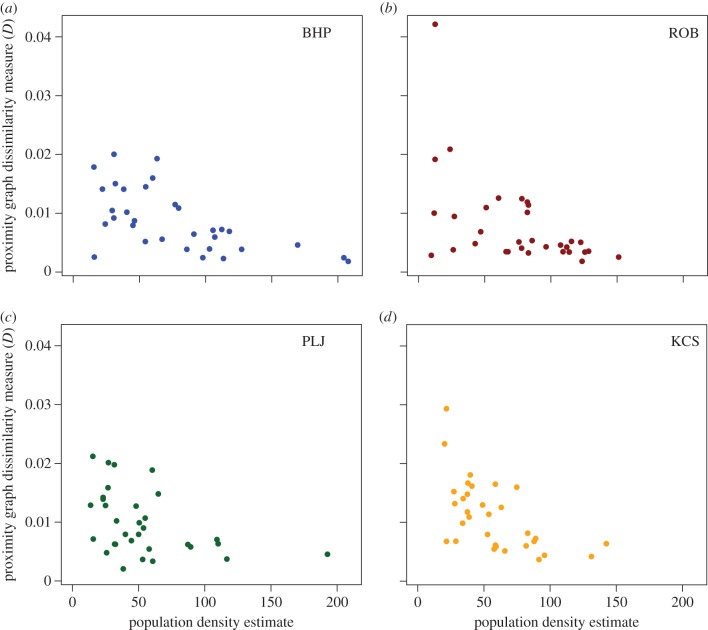
The results for the dissimilarity measure, *D*, quantifying the difference between an observed spatial graph and the family of proximity graphs based on the underlying point pattern. The values of *D* for the 32 networks from each site are plotted against estimated population density. The same pattern, that the observed graphs tend to become more similar to proximity graphs as network size increases, is replicated at each site. (Online version in colour.)

Figure 4 and electronic supplementary material, figure S9, show that for all four sites the dissimilarity measures, *D* and *D_u_*, tend to take lower values at higher vole population densities, indicating that the networks tended to become closer to proximity graphs. All four scatterplots have an absence of points in the upper right triangle of the axes, indicating that at higher densities vole contacts tended to be more like what would be expected on the basis of proximity alone: voles were more likely to interact with others closest to them, rather than being ‘selective’ in their contacts. However, while there was an absence of high values at high densities, there was no such absence of low values at low densities, indicating that at low population density some contact networks were very similar to proximity graphs and others were relatively dissimilar. The results for spatial variance quantified as distance deviation, a non-network measure of spatial restriction (see the electronic supplementary material), also show that voles reduce the spatial extent of their movements at higher densities (electronic supplementary material, figures S5 and S6). This is also consistent with the relationship between ɛ* and population density (see electronic supplementary material, figure S10) where again there is an absence of high values of ɛ* at high population densities.

### 3.4. Model fitting

**Figure 5.**
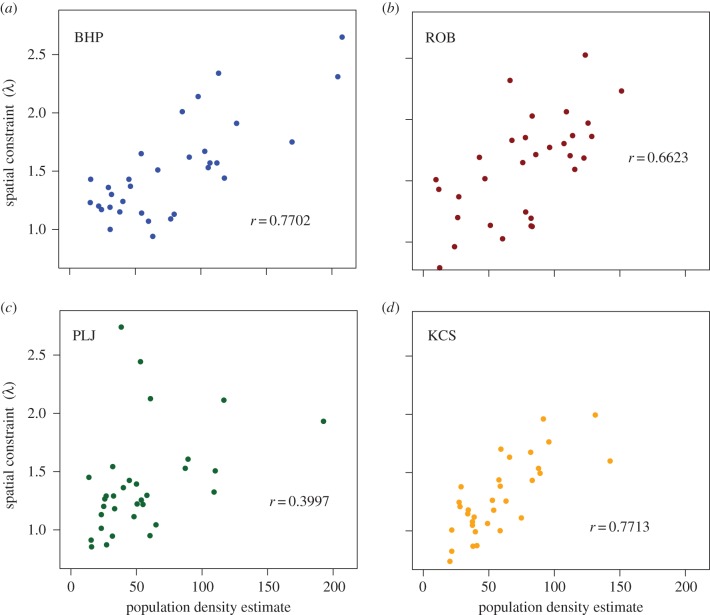
The model parameter *λ* (see Material and methods) which represents the strength of spatial constraints on contact rates between voles, were estimated for each of the 32 networks from each of the four sites and plotted against estimated population density. The results for the four sites are consistent and show a positive correlation. Pearson product–moment correlation coefficients are given as insets. (Online version in colour.)

The values of *λ*—obtained when the model given by equation (2.5) was fitted to each of the 128 networks—were positively correlated with vole population density, indicating once again that the spatial scale over which voles were likely to interact with others decreased with density (see figure 5). The relationship between *λ* and *D* is shown in electronic supplementary material, figure S8. The values of *λ* when the same statistical model that was fitted for the observed networks was fitted to 100 equivalent random networks were substantially less than the estimated *λ* value for the observed network: the average estimated *λ* for the equivalent random networks was 0.36 (s.d. 0.022) while the *λ* for the actual network was 1.69.

The results of the model fitting for the set of networks derived from site KCS and having at least 50 nodes are shown in table 1. The relative performance of the four models is measured using AICc. There is a uniformly sharp drop in AICc for model 1 compared with model 0, indicating strong support from the data for spatial proximity of nodes determining edges in these networks. For the majority of the networks, there was support for model 2 or 3 over model 1, and more often support for model 3 over model 2. Of the nine networks where model 1 had the greatest support, seven provided near-equivalent support for a more complex model (ΔAICc < 2): five for model 3 and two for model 2. Overall, therefore, there was support for the good-get-richer models that allowed large males, small males and females to differ in their overall propensity for contact (model 2) or in the degree to which distance discouraged contact (model 3). More often it was the latter. This overall picture was consistent across the four sites. When the data were pooled at the site level and a GLM similar to models 2 and 3 (see the electronic supplementary material) was fitted to the data all factors were highly significant, providing further evidence that the three categories of voles that we identified (female, small male and large male) are behaving differently and these behavioural differences do further explain the absence or presence of edges in the networks.

**Table 1.** AICc values for the four statistical models fitted to networks consisting of at least 50 individuals, for the site KCS. Model 0 represents the simplest case wherein the rate of contact is the same constant value (see equation (2.1)) for all pairs of individuals, equivalent to the random-mixing assumption. Model 1 represents a simple model wherein the rate of contact decreases with distance between individuals. Models 2 and 3 are similar, respectively given by equations (2.5) and (2.6) in the main text, both allow the parameters of Model 1 to vary between small males, large males and females, thus accounting for some heterogeneity in the vole population. The lowest AICc value is highlighted in boldface, as well as any other AICc values for which the difference in AICc from that for the best model is less than 2.

network	no. voles	model 0 (constant)	model 1 (spatial)	model 2 (spatial + heterogeneity1)	model 3 (spatial + heterogeneity2)
2	50	589	**327**	329	**327**
3	84	1105	**625**	628	**624**
4	65	1008	582	579	**571**
6	87	256	145	148	**137**
7	149	2459	1282	1279	**1275**
8	124	2825	1369	1358	**1352**
9	141	2621	**990**	**991**	**991**
10	125	1149	529	530	**525**
11	164	3124	1432	1407	**1386**
12	242	3527	1740	1726	**1718**
13	219	6659	3234	**3203**	3208
14	232	5208	**1970**	**1969**	**1969**
15	141	1967	770	768	**762**
18	64	403	202	**184**	187
23	96	614	339	**326**	335
24	141	2754	1589	1527	**1517**
25	107	2107	944	944	**935**
26	81	707	**334**	338	**336**
28	77	1272	742	**736**	740
29	98	407	**245**	**245**	252
30	135	1449	**856**	**854**	858
31	98	1043	**459**	461	**459**
32	69	518	**293**	295	296

When the edge data of all 128 networks was pooled the effect of density on the slope of the model and the effect of site on the intercept were both highly significant (see the electronic supplementary material). The results also show that the vole populations at two of the sites, ROB and BHP, perceive distance in more similar ways than any other pairing. Interestingly, the two similar sites are also those for which there was evidence that mean degree was a saturating function of population density.

Finally, the fitted good-get-richer model 3 indicated that for 19 of the networks large males were less discouraged by distance than either small males or females, and for the remaining four networks the small male class was the least discouraged by distance. On these same four occasions, the results for model 2 indicated that the small males had an overall greater propensity for contact. The female class was always the most discouraged by distance and almost always had the lowest propensity for contact (there were two exceptions).

## 4. Discussion

Overall, the results for our field vole system suggest that as population density increases, the mean numbers of contacts, 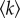, increases, and also that for at least two of the sites this increasing function saturates at the highest densities. In parallel with this, as density increases, voles are more likely to interact simply with those closest to them (lower values of *D* and *D_u_*) and the scale of spatial constraint increases (higher values of *λ*). We emphasize that *λ* and the graph dissimilarities measure different things—the parameter *λ* indicates the degree to which distance between voles acts as a deterrent for contact while *D* and *D_u_* indicate the similarity to a proximity graph without specifying that *ɛ* be small or large—though in this dataset *λ* and *D* are tightly related at higher densities and more loosely related for lower densities (see electronic supplementary material, figure S8). There was an absence of high values of *λ* at low values of vole density, indicating voles always took advantage of the ‘extra’ space, but this spreading out sometimes meant low values of *D* and sometimes not. At high vole densities though, the contact networks *always* became close to proximity graphs. They also became ‘tighter’ proximity graphs (lower values of ɛ*), with average distance deviation displaying much the same pattern as the other measures of spatial constraint.

The consistency of the relationships between the measures of spatial constraint and population density together paint a convincing picture that vole territories are shrinking with population density and becoming more strictly adhered to. The two dissimilarity measures *D* and *D_u_* both capture this effect. For this dataset, there in fact appears to be little difference between the two. Hence, while the underlying logic for the weighted version of the dissimilarity measure might be attractive, the additional complexity introduced by the weighting may be unnecessary.

The results here on contact rates increasing with density, but saturating at higher densities, are consistent with the findings of Smith *et al*. [18], and later Hu *et al*. [19], who worked with infection data from the same four natural populations of field voles over the same time period for cowpox virus, a pathogen transmitted by direct contact. We note that the contact networks we analyse are derived from trapping data and hence edges between individuals do not indicate direct contact, only that they shared the same trap at least once. The networks, therefore, are arguably most relevant to indirectly transmitted pathogens rather than directly transmitted pathogens such as cowpox. The correlation between indirect and direct contacts between voles is hard to predict and may depend on the age and sex of the animals involved. While two voles that share the same space are more likely to come into direct contact, behaviour will play an important role and some animals may actively avoid each other. However, the similarity between the results here and from studies of cowpox transmission may suggest that in this system there is a broad positive correlation between indirect and direct contacts, with both showing a similar relationship with density. Smith *et al*. [18] estimated the relationship between density and direct host contact rate by fitting the output of differential equation models to time-series data on cowpox virus infection. They concluded that the contact rate over the year as a whole is a saturating function of field vole density, best modelled as intermediate between density- and frequency-dependence.

Smith *et al.* [18] further noted that such nonlinearity is consistent with a variety of plausible mechanisms, such as heterogeneity in the host–contact network (with a higher proportion of low-contact hosts at higher densities), or the limiting time available for contacts to be made (such that contact rate cannot keep pace with increasing density), or simply changes in the behaviour of individuals with population density (e.g. with individuals becoming more territorial at higher densities, and only contacting those on territory borders). One key benefit of studies such as those conducted here is that they may shed light on the mechanistic (individual-level) basis for these population-level phenomena. In the present case, heterogeneity in contact rate decreased rather than increased at higher densities and so is unlikely to have contributed to the saturating curve. Our analysis adds nothing to considerations of time limitation, but we believe that the nature of vole contacts, passing one another in shared runways in the grass, itself makes it unlikely that they ever reach a position where there is simply no more time for further contact with conspecifics. The tendency, however, for voles to make contacts throughout the population at lower densities does indeed make it likely, when density is low, that contact rate will increase with density: the classic basis for density-dependent transmission [25]. Whereas space-constrained contacts at higher densities are akin to territorial behaviour and the consequent tendency to contact only territorial neighbours, whose numbers are relatively independent of density overall (classic frequency-dependence). Our results therefore suggest that the transmission function lying ‘between’ density- and frequency-dependence selected by the analysis in Smith *et al*. [18] is generated by contacts being closer to density-dependence at low densities and closer to frequency-dependence at high densities.

Even so, while a transition to frequency-dependence might be a good description of how the *numbers* of contacts changes with density, the tendency to contact only territorial neighbours implies that the *spatial distribution* of contacts also changes with density. At higher densities then, there is a more severe departure from the random-mixing assumption that underpins the differential equation models used by Smith *et al*. [18]. As spatial constraints on contacts increase there will be a stronger local saturation effect: infected individuals will be more often surrounded by recovered or already infected individuals. Hence, part of the explanation for the ‘transitioning’ phenomena at the population level may be that the fitted differential equation models underestimate the contact rate (as a function of density) in order to avoid overestimating the force of infection on the remaining susceptible portion of the population.

As well as the mean number of contacts increasing with population density, we observed that the level of individual variation in contact rate also decreases. It is well known that the variance of the degree distribution of a contact network enters calculations for the basic reproduction number in a nonlinear way, and that
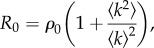
where *ρ*_0_ is defined to be the basic reproduction number if there was no heterogeneity in the numbers of contacts, and where the sharp brackets represent averages over the degree distribution, *P*(*k*) [9]. Note that this equation does not take into account the effects of any clustering in the network and the quantity 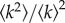 is the CV of the degree distribution. For the field vole populations, the data show that *ρ*_0_ increases with density (owing to increases in the average number of contacts) and hence *R*_0_ too, but two other things also happen that would reduce *R*_0_ or reduce the force of infection on the susceptible part of the population: (i) the CV decreases (the networks become increasingly homogeneous), and (ii) the networks become more spatial. Hence, our data imply that there may be a cancellation effect for *R*_0_, and more generally for pathogen transmission, whereby an increase in contact rate (and hence increase in transmission) owing to higher densities is at least partially ‘cancelled out’ by a decrease in individual heterogeneity and an increase in the spatial constraints on the contacts of the voles. This would predict that the spread of pathogens on these vole contact networks could be relatively insensitive to fluctuations in host density, and hence suggests an additional hypothesis as to why abundance thresholds operating in wildlife disease systems are rarely detected [26].

The good-get-richer models which divided the population into a large male class, a small male class and a female class were broadly supported by the vole data and suggested mature males were more likely to make contact with conspecifics than other groups of voles. The four networks (from KCS) for which this was not true show that the small male class instead had the greatest propensity for contact and was less constrained by distance. These networks may correspond to trapping at times of the year where young males are dispersing and seeking to establish their own territory. Overall, these patterns add to the growing number suggesting that large males may be particularly important in the transmission of infection because they are ‘super-contactors’ (e.g. [7]).

Data for determining animal contact networks are collected through a wide range of techniques (for a review see [3]), but most often animals must be captured and tagged, meaning that the contacts observed are between animals based within the same finite area. Hence, datasets will inevitably exclude long-range contacts that arise, for example, from dispersal movements of maturing animals, and are unlikely to include contacts with nomadic individuals moving through the study area, tending to overestimate spatial constraints. However, Craft *et al*. [2] concluded that for the network of Serengeti lions, the presence of nomadic individuals had marginal epidemiological impact, especially for pathogens with short infectious periods. This supports our contention that even with rare long distance events, spatial analyses of contact networks are worthwhile and spatial constraints may play an important role.

The results here suggest that we stand to gain three things from constructing well-defined network measures of spatial constraint. First, we are able to infer that our animal contact networks are indeed spatial. Values of *D* and *λ* can be compared with networks from randomized equivalent trapping data to show that the observed network has significantly higher values (it is necessary to randomize the trapping data, rather than the edges of the observed network, if trap locations are used to allocate individuals a spatial position *and* define edges). Second, we gain a means of quantifying differences between two or more contact networks and so a basis for comparing different sites, species or times of year. Third, the measures lay the basis for mathematical descriptions of wildlife contact networks which could be used to generate theoretical networks representing host populations at much larger spatial scales, more relevant to real wildlife populations.

## Supplementary Material

Electronic Supplementary Material for “Spatial analyses of wildlife contact networks”

## Data accessibility

The authors are happy to make the original data available to others. Contact either M.B. (mbegon@liv.ac.uk) or S.T. (s.telfer@abdn.ac.uk).
