# Supplementary material for: Spatial analyses of wildlife contact networks
Source: J R Soc Interface. 2015 Jan 6;12(102):20141004. doi: 10.1098/rsif.2014.1004 (PMC4277090; doi:10.1098/rsif.2014.1004)
Supplement: Electronic Supplementary Material for “Spatial analyses of wildlife contact networks” [file rsif20141004supp1.pdf]

Electronic Supplementary Material for "Spatial analyses of wildlife contact networks"

S1. Contact networks

The 32 contact networks from one field site, Keilder Central Site (KCS), are shown in Figure S1 as non-spatial graphs (where connectedness is clearly visible) and in Figure S2 as spatial graphs (as they were treated when fitting various statistical models). The 128 networks we analysed are based on combining trapping data from pairs of consecutive sessions. An alternative, as discussed in the main text, is to construct networks by combining data from 3 consecutive trapping sessions. The result is a set of 21 networks for each site. For comparison purposes the resulting networks from KCS are shown as non-spatial graphs (Figure S3) and spatial graphs (Figure S4).

S2. Distance deviation analysis

The spatial variance at the individual vole scale, observed as the spatial spread of the trap locations at which an individual vole was caught, was quantified using a distance deviation for every node (vole) in each of the 128 networks we analysed. This is calculated based on the one or more trap locations as,

$$d = \sqrt{\frac{\sum_{i=1}^{N}(x_i - \bar{x})^2 + (y_i - \bar{y})^2}{N}}$$

where *N* is the number of trap locations an individual vole was caught at during the two consecutive trapping sessions. This takes on a value of 0 if the vole was caught only once, or if the vole was repeatedly caught in the same trap.

Our first interest in this individual measure was to examine how the distribution of the distance deviation changed with the density of the vole population. To do this we plotted the data (Figure S5) such that each point is a transparent disc with area equal to the number of voles having that individual measure of distance deviation. Hence, one can see the distributions of distance deviations against vole density for the 32 networks for each field site. We also calculated average distance deviation for each site, each network and each category of vole (female, large male, small male) and plotted these again population density (Figure S6). The average distance deviation shows the same trends as network measures of spatial constraint: the voles shrink the spatial extent of their movements as densities become higher.

Next we examined if there was any relationship between the category-specific lambda values estimated using model 3 and the expected distance deviation for that category. The scatter plot for the two variables is shown in Fig S7 for the 32 networks derived from trapping data collected at KCS. The two measures of spatial movement are negatively correlated (females, r = -0.3293; males, r = -0.4697; large males, r = -0.6173), which is as expected since one is a measure of inhibition and the other is a measure of freedom. The spread of average distance deviations is larger for the large males and the strongest correlation is for this category.

S3. Statistical modelling

When using generalized linear models for model 0, we fit y ~ 1, and for model 1 we fit y ~ d where d is the Euclidean distance between the pair of nodes. The AICc values for all 32 networks from KCS for models 0, 1 and 3 are shown in Table S1 together with the results for a fourth model, y ~ (edge type)*d, where “*” represents interaction and “edge type” is a categorical variable for the six possible pairings of vole categories (female-female, female-small male, female-large male, small male-small male, small male-large male, large male-large male). Model 4 is similar to models 2 and 3 in the main text because the interaction allows the 6 different pairings to affect the intercept (similar to model 2) and slope (similar to model 3) of the linear model. Model 4 is different to both model 2 and 3 because model 2 only allows the pairing type to affect the intercept and model 3 only allows the pairing type to affect the slope. This model was found to give similar results to models 2 and 3. Whether a drop in AIC was observed was mixed. The generalized linear model approach sometimes did not give a lower AIC compared to model 1 even though the maximum likelihood method did and vice versa.

Next we combined all 128 networks and tested the effects of vole population density and study site variables on the slope and intercept. The model y ~ d + site + N where site={BHP, KCS, PLJ, ROB} was fitted to the data where site is a categorical variable. All factors were highly significant and hence site affects the intercept and population density affects the slope. The model fit from the glm function in R was as follows:

**Variable**	**Estimate**	**Std. Error**	**z value**	**Pr(>|z|)**
(Intercept)	1.5052893	0.0229196	65.677	< 2e-16
d	-1.5133823	0.0070164	-215.691	< 2e-16
N	-0.0037631	0.0001107	-33.999	< 2e-16
KCS	0.2268413	0.0180017	12.601	< 2e-16
PLJ	0.2214788	0.0174525	12.690	< 2e-16
ROB	-0.1037824	0.0167206	-6.207	5.41e-10.

The significance of ROB is indicative that for two of the sites, ROB and BHP, the intercepts and slopes are similar, suggesting the voles in those two populations perceive distance in similar ways. The simpler model y ~ d*site confirmed that these two sites are more similar to each other than any other pairing. In this case the model fit from the glm function in R was as follows:

**Variable**	**Estimate**	**Std. Error**	**z value**	**Pr(>|z|)**
(Intercept)	0.97749	0.02060	47.454	< 2e-16
d	-1.56314	0.01290	-121.180	< 2e-16
KCS	0.16322	0.03202	5.098	3.44e-07
PLJ	0.14618	0.03221	4.538	5.67e-06
ROB	0.06873	0.03070	2.239	0.0251
d:KCS	0.17114	0.01882	9.091	< 2e-16
d:PLJ	0.08228	0.01961	4.195	2.73e-05
d:ROB	-0.04214	0.01957	-2.153	0.0313.

Finally, the data were also pooled at the individual site level and the model y ~ (edge type)*d + N fitted, where N is the population density of voles at the time an edge was observed. Again, all factors were highly significant, confirming that the three categories of voles are affected by distance differently and that these behavioral differences can further explain the absence or presence of edges in the networks.

Table S1. Akaike's Information Criterion values, corrected for finite sample sizes, for the 32 networks inferred from trapping at KCS, and for 6 models, the first 3 being fitted using maximum likelihood techniques and the second 3 being fitted using the generalized linear modeling approach.

**Network**	**Model 0 (y ~ 1)**	**Model 1 (y ~ d)**	**Model 3 (ML)**	**Model 4 (y ~ d*(edge type))**
1	711	417	416	413
2	590	327	327	339
3	1105	625	624	629
4	1008	582	571	576
5	428	228	229	240
6	256	145	137	148
7	2459	1282	1275	1278
8	2825	1369	1352	1372
9	2621	990	991	983
10	1149	529	525	531
11	3124	1432	1386	1384
12	3527	1740	1718	1713
13	6659	3234	3208	3190
14	5208	1970	1969	1963
15	1967	770	761	772
16	669	366	371	376
17	342	153	157	157
18	403	202	187	200
19	976	526	534	535
20	307	187	203	197
21	84	52	58	61
22	207	145	158	147
23	614	339	335	317
24	2754	1589	1517	1521
25	2107	944	935	945
26	707	334	336	330
27	368	193	198	196
28	1272	742	740	742
29	407	245	252	244
30	1449	856	858	862
31	1043	459	459	466
32	518	293	296	297

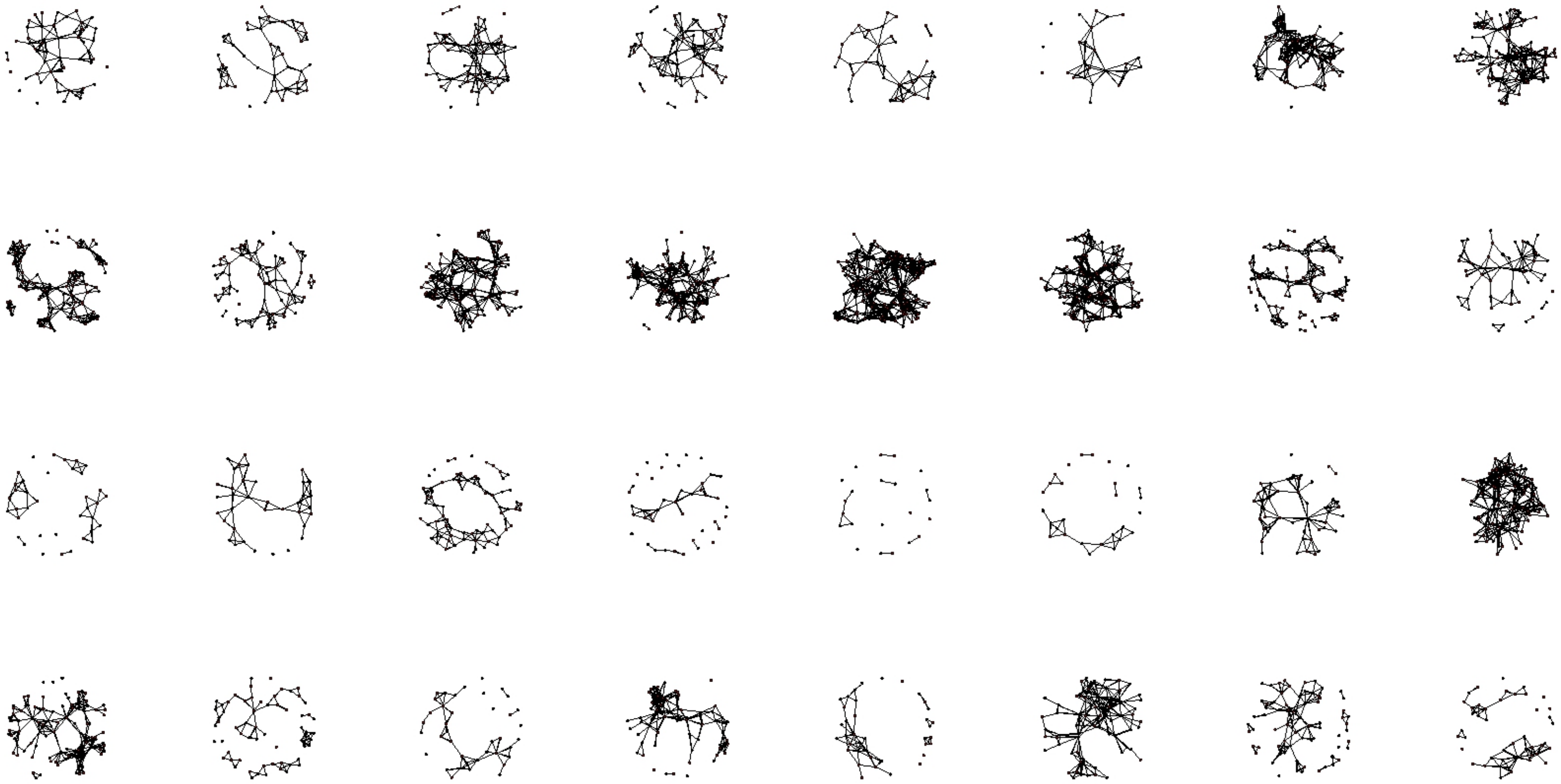

Figure S1.

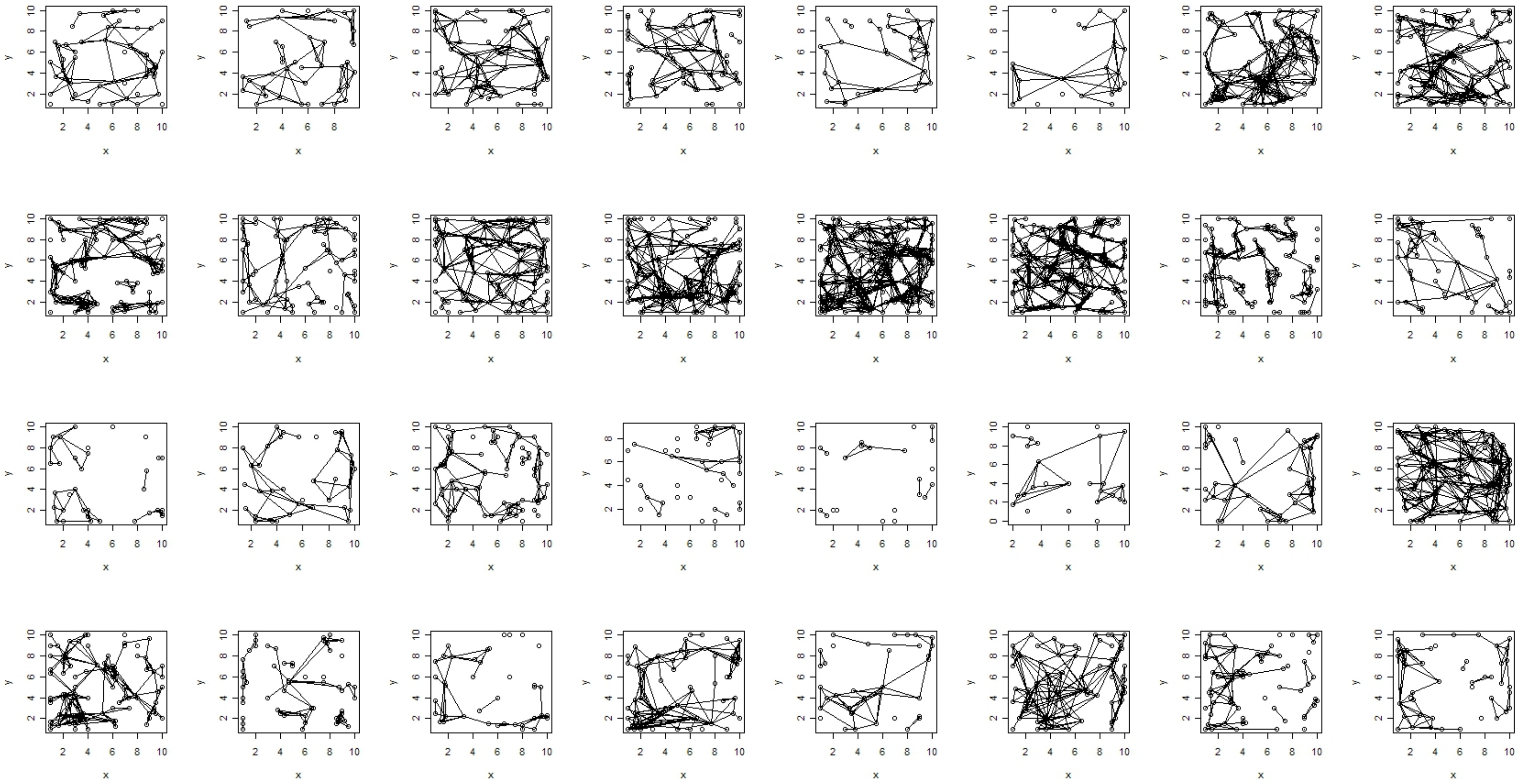

Figure S2.

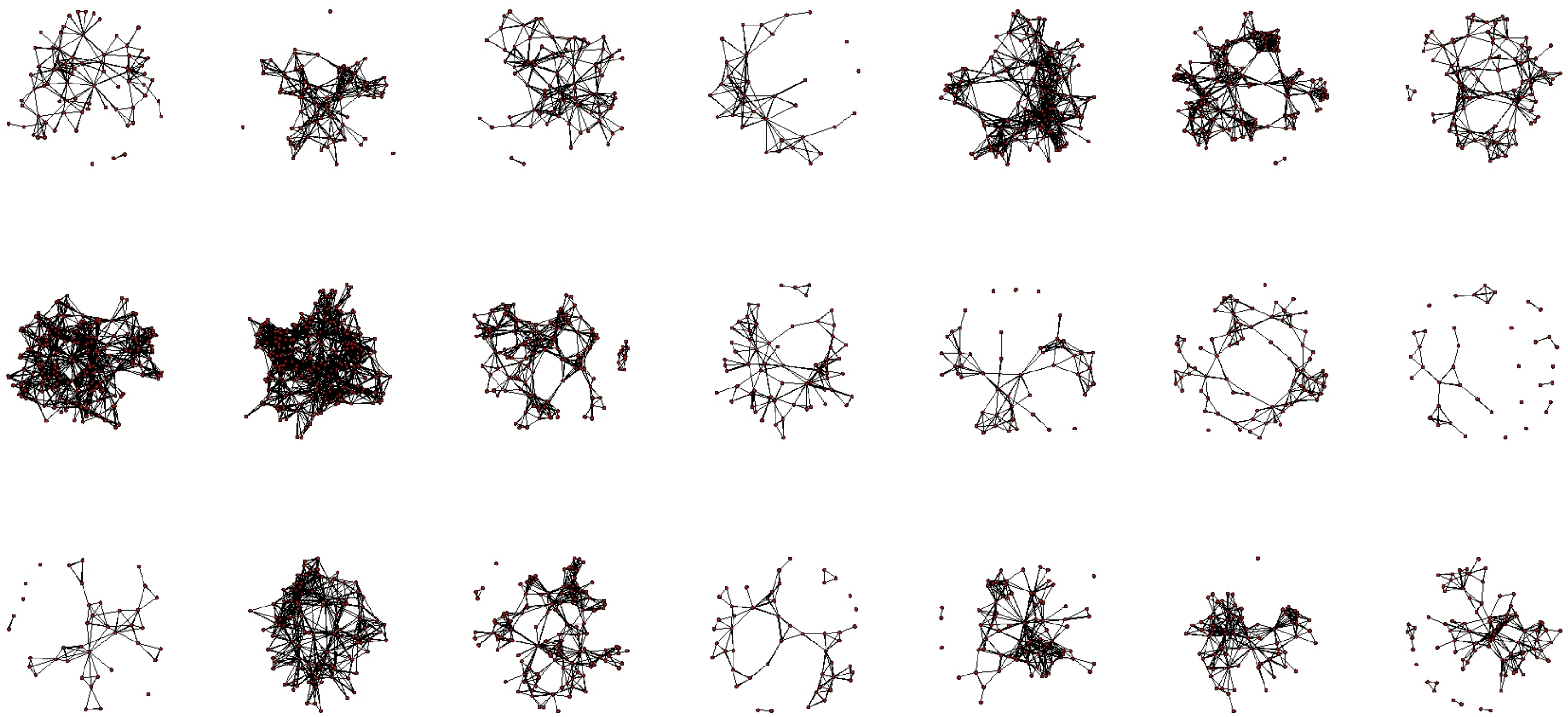

Figure S3.

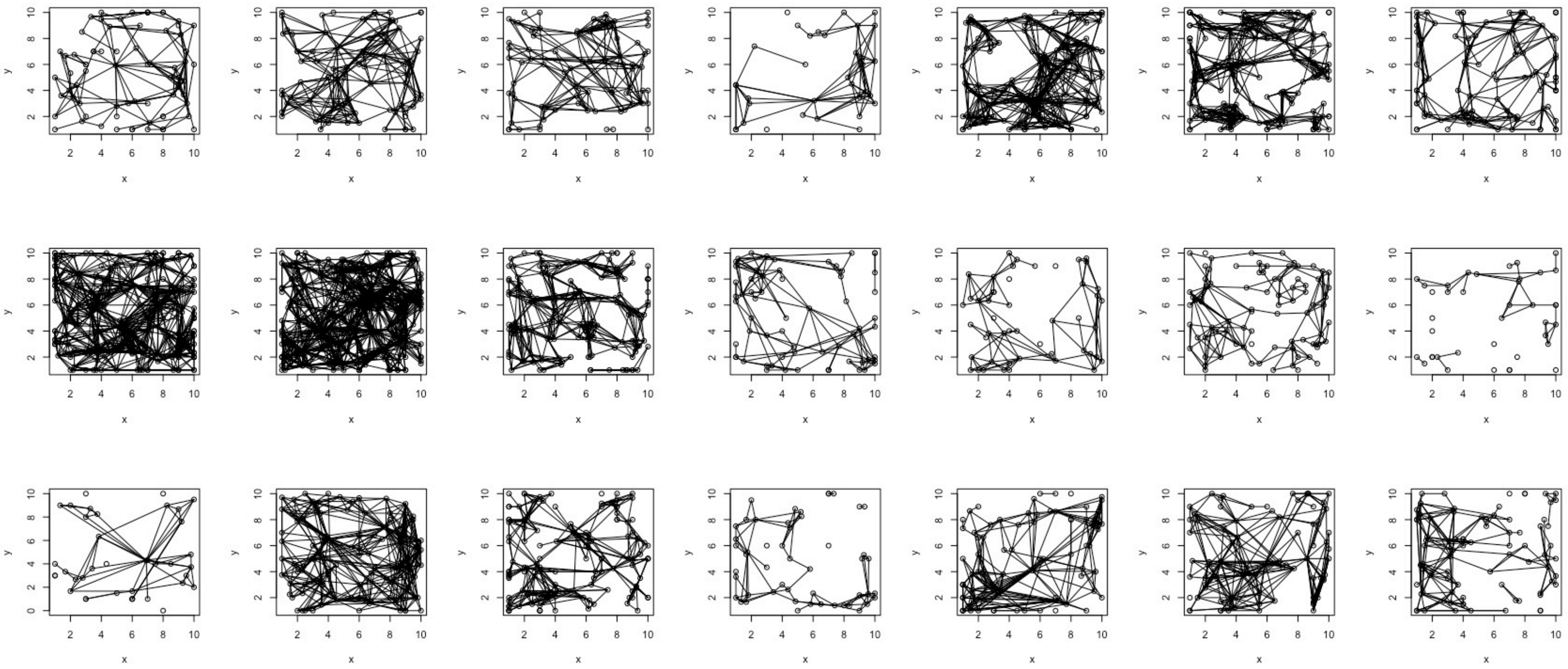

Figure S4

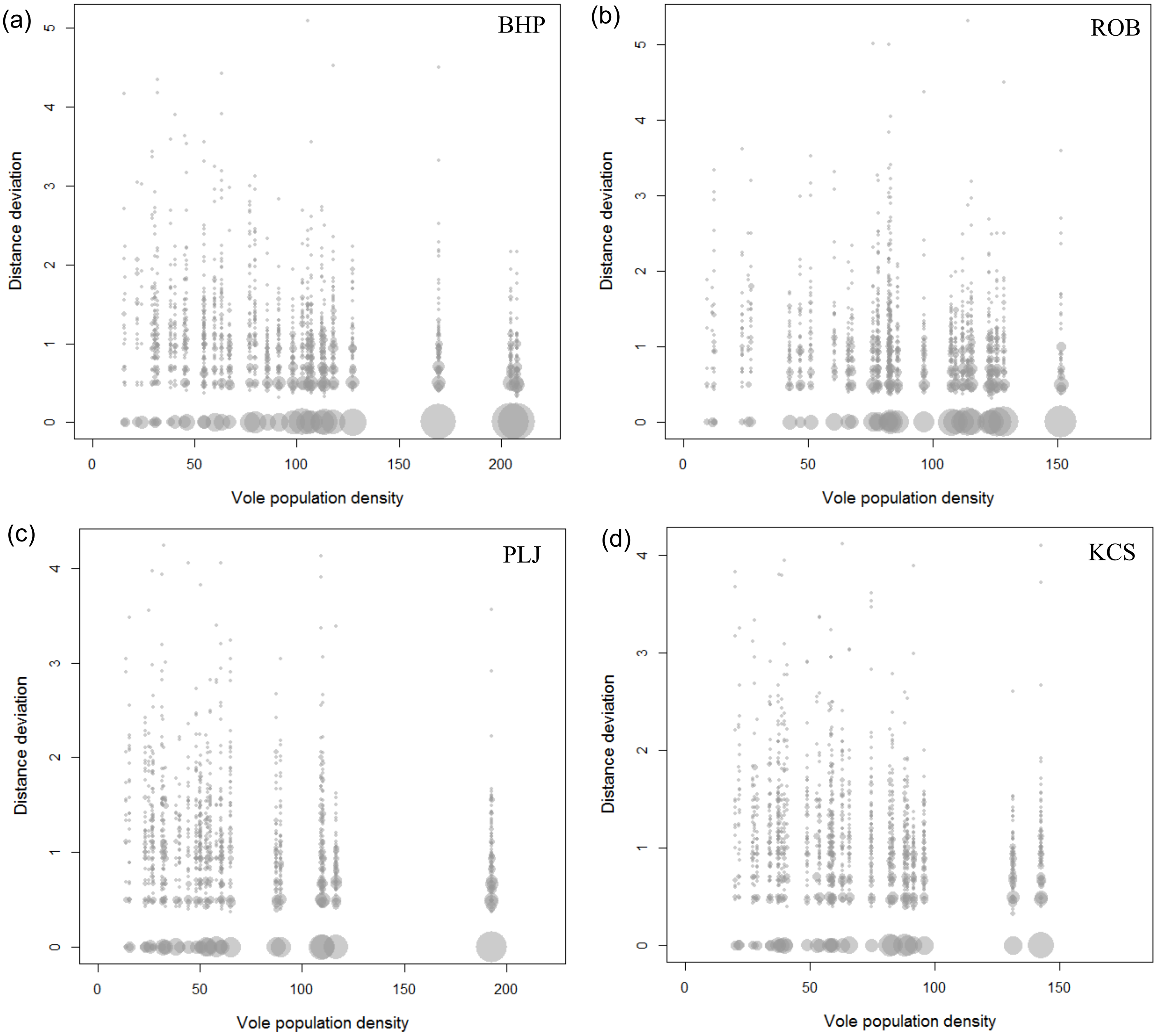

Figure S5. Individual distance deviation versus vole population density where area is proportional to the number of voles.

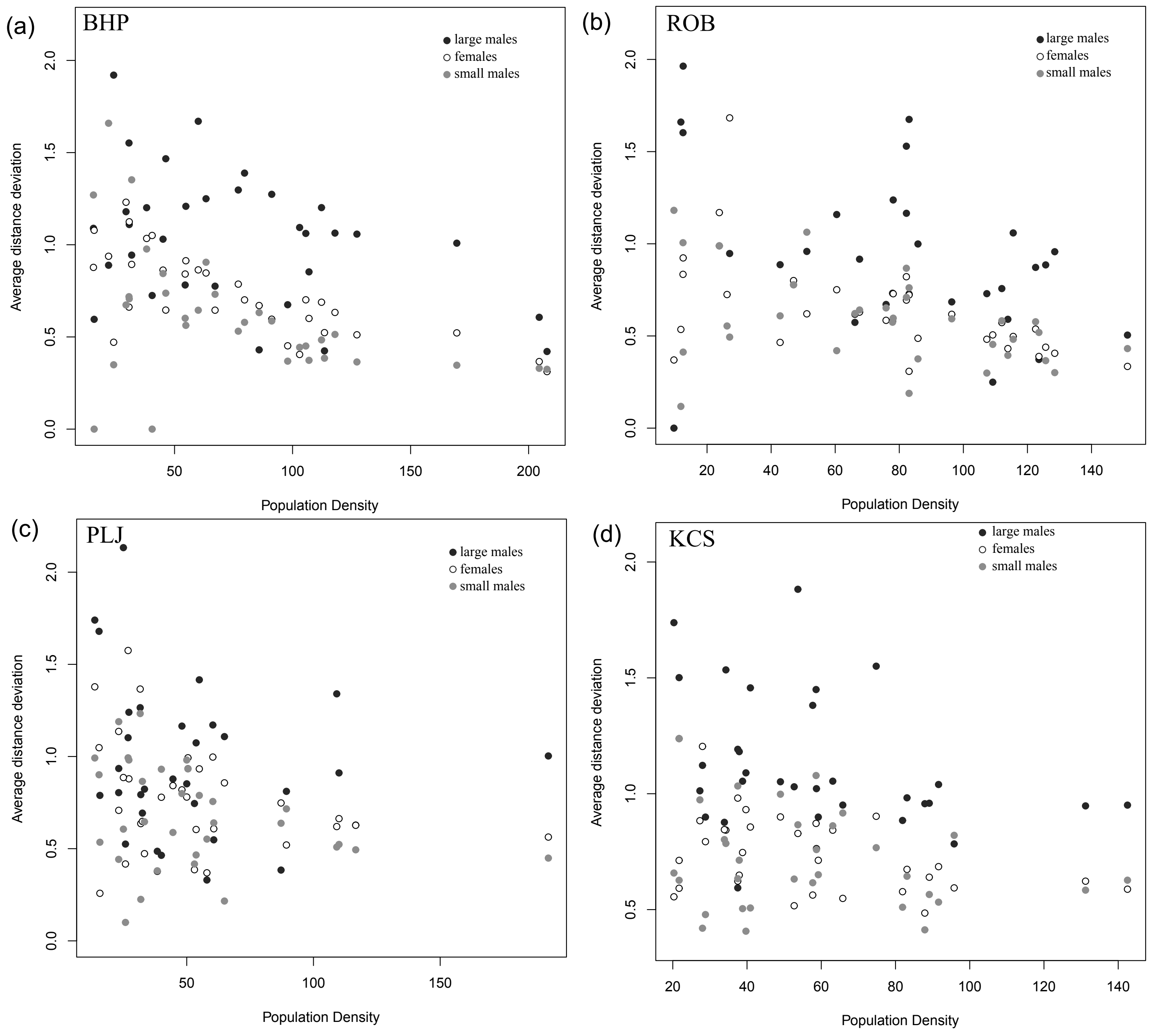

Figure S6. Average distance deviation for each category of vole – large male, female and small male – calculated for each of the 128 networks and plotted against vole population density.

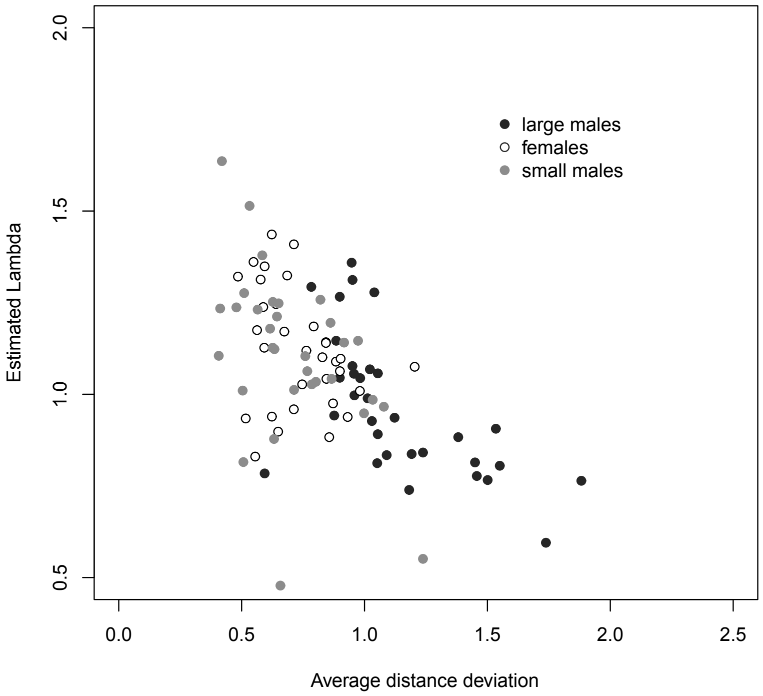

Figure S7. Estimated lambda values plotted again the average distance deviation for the 32 networks formed from the trapping data at one site (Keilder Central Site) and for the three categories of vole.

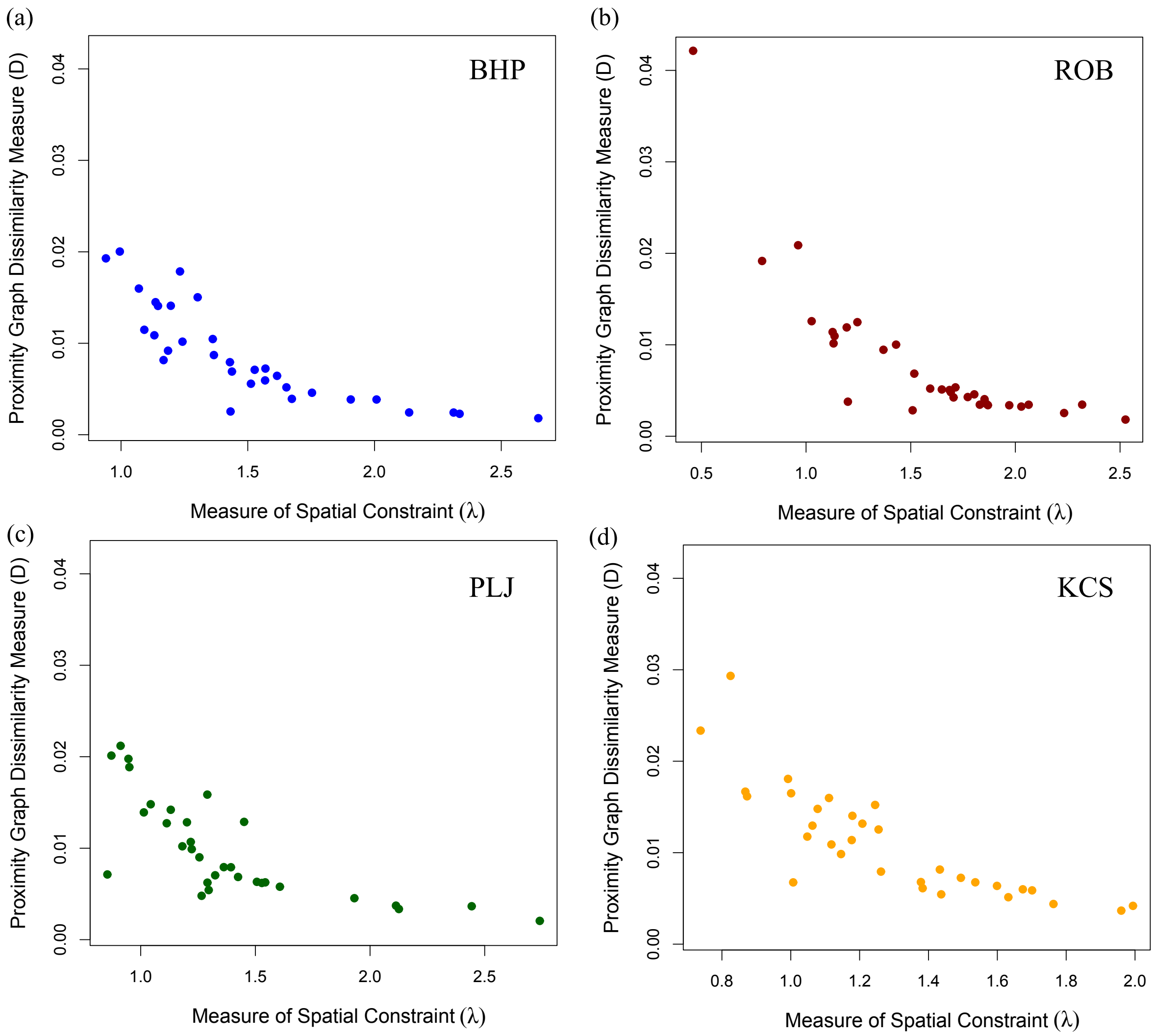

Figure S8. Estimated lambda values plotted against the graph dissimilarity measure *D* calculated for each of the 128 networks.

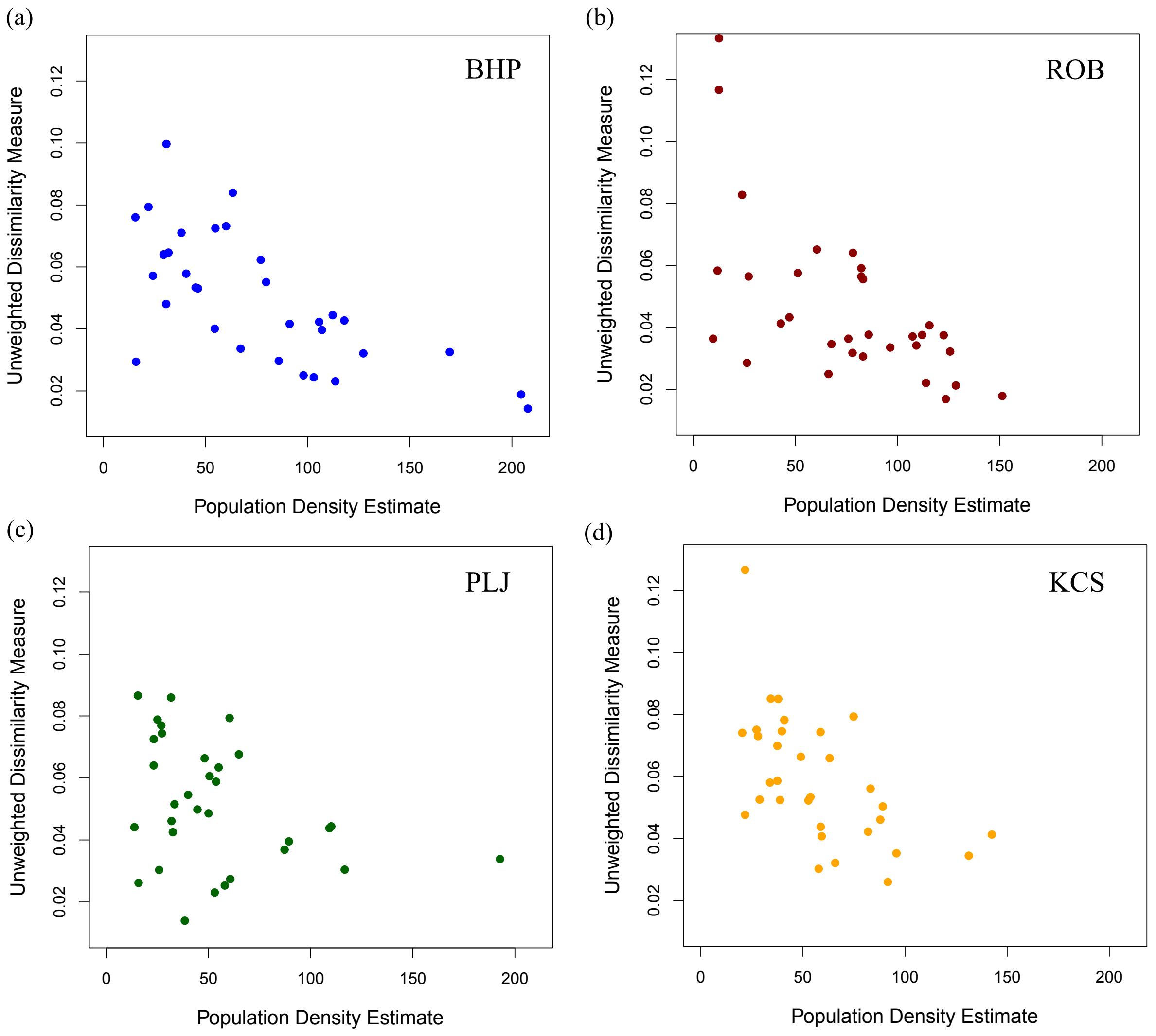

Figure S9. The unweighted graph dissimilarity measure D_u calculated for each of the 128 networks and plotted against vole population density.

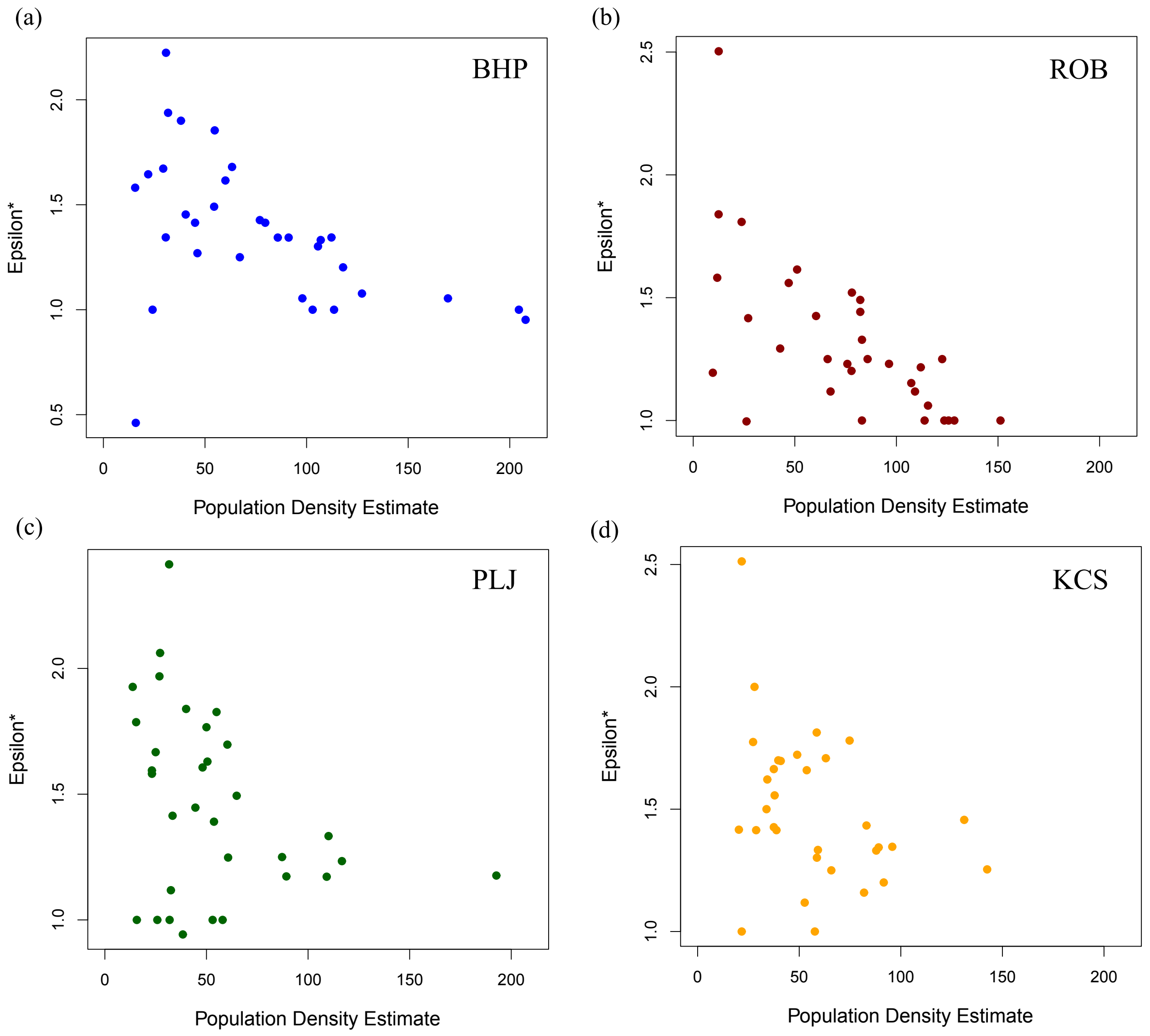

Figure S10. The value ε^* calculated for each of the 128 networks when determining the graph dissimilarity measure D, plotted against vole population density.
